# Platelet and myeloid lineage biases of transplanted single perinatal mouse hematopoietic stem cells

**DOI:** 10.1038/s41422-023-00866-4

**Published:** 2023-09-06

**Authors:** Karin Belander Strålin, Joana Carrelha, Axel Winroth, Christoph Ziegenhain, Michael Hagemann-Jensen, Laura M. Kettyle, Amy Hillen, Kari Högstrand, Ellen Markljung, Francesca Grasso, Masafumi Seki, Stefania Mazzi, Yiran Meng, Bishan Wu, Edwin Chari, Madeleine Lehander, Rickard Sandberg, Petter S. Woll, Sten Eirik W. Jacobsen

**Affiliations:** 1 Department of Medicine Huddinge, Center for Hematology and Regenerative Medicine, Karolinska Institutet, Stockholm, Sweden; 2 Department of Pediatric Oncology, Karolinska University Hospital Solna, Stockholm, Sweden; 3 Haematopoietic Stem Cell Biology Laboratory, MRC Weatherall Institute of Molecular Medicine, University of Oxford, Oxford, UK; 4 MRC Molecular Haematology Unit, MRC Weatherall Institute of Molecular Medicine, University of Oxford, Oxford, UK; 5 Department of Cell and Molecular Biology, Karolinska Institutet, Stockholm, Sweden; 6 Karolinska University Hospital Huddinge, Stockholm, Sweden

**Keywords:** Haematopoietic stem cells, Haematopoietic stem cells

Dear Editor,

Hematopoietic stem cells (HSCs) residing in adult mouse bone marrow (BM) are highly heterogenous,^1^ ranging from HSCs executing their full multilineage potential to those with platelet (P)-biased/restricted output.^2–4^ These distinct lineage-biased propensities are sustained upon secondary transplantation, and thus largely intrinsic.^1^ While adult HSCs derive from definitive fetal HSCs,^5^ it is unclear to what degree the lineage biases/restrictions observed in adult BM HSCs, or any other distinct lineage biases/restrictions, have been established before birth.

Definitive HSCs expand in the mid-gestation fetal liver, and at embryonic day (ED) 17.5, they start migrating to the BM, the primary hematopoietic site throughout life.^5^ Previous transplantation studies of single fetal HSCs, assessing only white blood cell lineages, identified myeloid (M)-biased and lymphoid (L)-biased HSCs in ED14.5 and ED18.5 liver.^6^ Whereas previous studies identified P-biased/restricted adult HSCs,^2–4^ the propensity of fetal and perinatal HSCs (pnHSCs) to replenish the critical platelet and erythroid (E) lineages has yet to be investigated at a clonal level in mice or humans. Herein, we systematically analyzed post-transplantation five-lineage blood replenishment by single pnHSCs at ED19.5/postnatal day 0 (PD0). Utilizing a *Vwf*-tdTomato^tg/+^;*Gata1*-eGFP^tg/+^ mouse transplantation model,^2^ we assessed clonal contribution over time to blood P, E, M, B and T cells by single Lineage^−^Sca1^+^Kit^+^(LSK)CD150^+^CD48^−^ HSCs from liver and BM (Fig. 1a; Supplementary information, Fig. S1 and Table S1). 22% of mice transplanted with a single liver or BM pnHSC showed ≥ 0.1% long-term (LT) contribution to at least one blood cell lineage after 25–26 weeks (Supplementary information, Fig. S1a). Over 90% of reconstituting single pnHSCs (from liver or BM, Supplementary information, Fig. S1) replenished all blood lineages (Fig. 1a), in contrast to < 50% of single adult BM LT-HSCs.^2^ Whereas a third of single adult LT-HSCs contribute exclusively to PEM lineages,^2^ no pnHSCs were P- or PEM-restricted (Fig. 1a, b) in liver or BM (Supplementary information, Fig. S1b). Likewise, whereas half of adult LT-HSCs possess P/PEM-bias,^2^ only one pnHSC from BM and none from liver displayed P-bias, and < 15% displayed PEM-bias (Fig. 1c; Supplementary information, Fig. S1c). A small fraction of pnHSCs were PEMB-restricted at 25–26 weeks (Fig. 1a, b), representing the only lineage restriction of pnHSCs at this time. However, while most P- and PEM-restricted adult HSCs remain restricted in primary recipients,^2^ for most pnHSCs showing PEMB-restricted reconstitution at 25–26 weeks, T cell output became apparent with time (Supplementary information, Fig. S2a, b). Thus, overall, transplanted pnHSCs show less P- and PEM-bias/restriction and more consistent lymphoid output than adult HSCs.

**Fig. 1:**
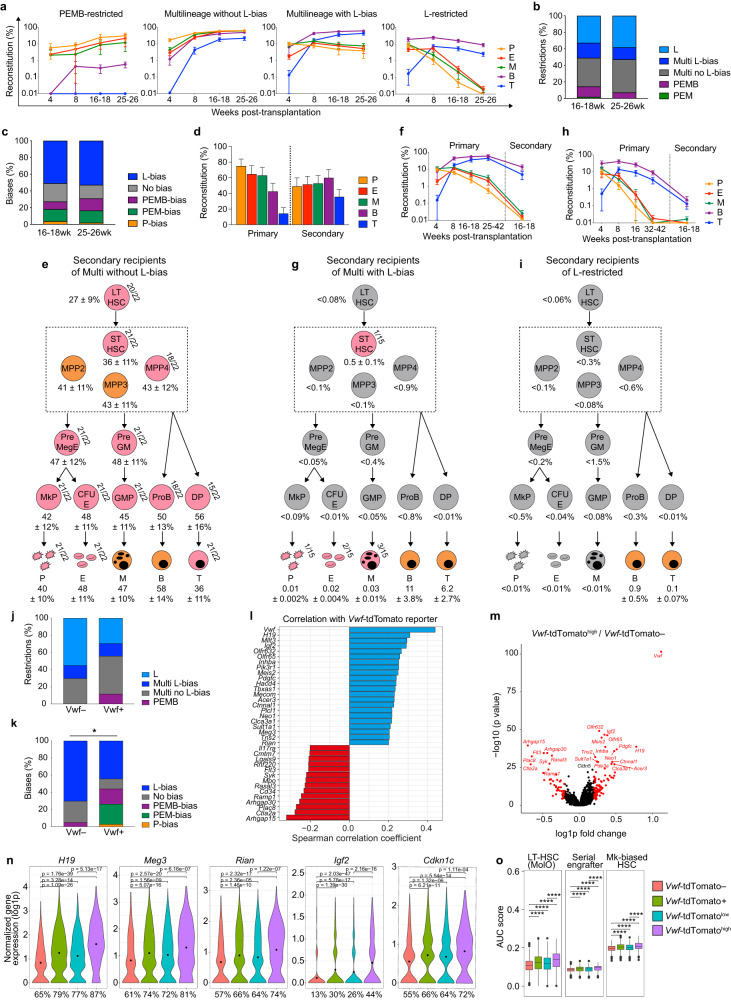
Reconstitution patterns of pnHSCs. **a** LT blood reconstitution patterns upon primary transplantation of a single LSKCD150^+^CD48^−^ pnHSC sorted from mouse liver or BM on ED19.5/PD0 (*n* = 252 recipients; 7 independent experiments). Mean ± SEM for each lineage. PEMB-restricted, *n* = 4; multilineage without L-bias, *n* = 22; multilineage with L-bias, *n* = 8; L-restricted, *n* = 21. Note that all L-restricted patterns showed replenishment of PEM lineages at earlier time points. **b**, **c** Lineage restrictions (**b**) and biases (**c**) among reconstituted mice at 16–18 weeks and 25–26 weeks post-primary transplantation (*n* = 55). Lineage-restricted patterns were defined by undetectable (<0.01%) contribution to one or more blood lineage. Biased patterns are defined in the Supplementary Methods. **d**, **e** Blood replenishment (mean ± SEM) in primary recipients of a single pnHSC with multilineage reconstitution without L-bias at the time of secondary transplantation (*n* = 12, 26–42 weeks post-primary transplantation) and in corresponding secondary recipients (*n* = 12, primary donors, 1–3 secondary recipients per donor, 16–18 weeks post-secondary transplantation, (**d)**), and replenishment (mean ± SEM) of HSPCs in secondary recipients (*n* = 8, primary donors, 1–3 secondary recipients per donor, 17–27 weeks post-secondary transplantation (**e**)). **f**, **g** Blood replenishment (mean ± SEM) in primary recipients of a single pnHSC with multilineage reconstitution with L-bias at the time of secondary transplantation (*n* = 7, 25–49 weeks post-primary transplantation) and in corresponding secondary recipients (*n* = 7, primary donors, 1–3 secondary recipients per donor, 16–18 weeks post-secondary transplantation (**f**)), and replenishment (mean ± SEM) of HSPCs in secondary recipients (*n* = 7, primary donors, 1–3 secondary recipients per donor, 17–28 weeks post-secondary transplantation (**g**)). **h**, **i** Kinetics of blood replenishment (mean ± SEM) in primary recipients of a single pnHSC with L-restricted reconstitution at the time of secondary transplantation (*n* = 6, 32–42 weeks post-primary transplantation) and in corresponding secondary recipients (*n* = 6, primary donors, 2–3 secondary recipients per donor, 16–18 weeks post-secondary transplantation (**h**)), and replenishment (mean ± SEM) of HSPCs in secondary recipients (*n* = 3, primary donors, 1–3 secondary recipients per donor, 27 weeks post-secondary transplantation (**i**)). Grey denotes populations with no detectable donor contribution in any recipients, pink denotes contribution in some but not all recipients (frequency of reconstituted mice specified), and orange denotes contribution in all recipients (**e**, **g**, **i**). PreMegE, pre-megakaryocyte/erythroid progenitor; MkP, megakaryocyte progenitor; CFU-E, colony forming unit-erythroid; PreGM, pre-granulocyte/monocyte progenitor; GMP, granulocyte/monocyte progenitor; DP, double positive T cell progenitors. **j**, **k** Lineage restrictions (**j**) and biases (**k**) of single pnHSCs at 25–26 weeks post-primary transplantation, re-analyzed based on *Vwf*-tdTomato expression (*Vwf*-tdTomato^−^, *n* = 20; *Vwf*-tdTomato^+^, *n* = 34). No statistically significant association between variables with Fisher’s exact test in **j** (*P* = 0.1679), but statistically significant in **k** (*P = 0.0259). **l** Spearman correlation analysis of single pnHSCs (*n* = 1700) for gene expression associated with *Vwf*-tdTomato level determined by FACS index-sort. Genes with positive (blue; correlation coefficient > 0.2) and negative (red; correlation coefficient < −0.2) correlation with *Vwf*-tdTomato expression are shown. **m** Volcano plot for DEGs (red; combined *P* < 0.05) when comparing single *Vwf*-tdTomato^high^ (*n* = 283) with *Vwf*-tdTomato^−^ (*n* = 1128) pnHSCs. Downregulated genes on the left (log1p fold change < −0.2) and upregulated genes on the right (log1p fold change > 0.2). **n** Normalized expression (log1p) for selected DEGs (combined *P* < 0.05) in single *Vwf*-tdTomato^−^ (*n* = 1128), *Vwf*-tdTomato^+^ (*n* = 572), *Vwf*-tdTomato^low^ (*n* = 289), and *Vwf*-tdTomato^high^ (*n* = 283) pnHSCs. Mean expression is represented by dots within violin plots and percentage of cells with detected expression is indicated below. **o** AUC scores for LT-HSC (“molecular overlap”, MolO), serial-engrafter HSC, and Mk-biased HSC signatures for single *Vwf*-tdTomato^−^ (*n* = 1128), *Vwf*-tdTomato^+^ (*n* = 572), *Vwf*-tdTomato^low^ (*n* = 289), and *Vwf*-tdTomato^high^ (*n* = 283) pnHSCs. Wilcoxon rank sum test. ****P < 0.001.

Many multilineage pnHSCs showed no prominent lineage bias (Fig. 1c), sustaining LT production of all blood lineages, and replenishing hematopoietic stem and progenitor cells (HSPCs) in primary and secondary recipients (Fig. 1d, e; Supplementary information, Fig. S2c–g), confirming their extensive self-renewal and stable lineage bias.

In agreement with published ED18.5 data,^6^ many pnHSCs showed L-biased and L-restricted LT contribution to blood (Fig. 1a–c; Supplementary information, Fig. S1b, c). However, L-bias of multilineage-reconstituting LSKCD150^+^CD48^−^ cells only became apparent weeks or months post-transplantation, and all L-restricted cases contributed to PEM lineages at earlier time points (Fig. 1a). The L-restricted contribution was typically preceded by L-biased reconstitution (Fig. 1a; Supplementary information, Fig. S3a, b), and primary L-biased reconstitution patterns became L-restricted upon secondary transplantation, with drastically reduced PEM reconstitution (Fig. 1f–i). HSPC replenishment was low and inconsistent in primary recipients of L-biased/restricted HSCs (Supplementary information, Fig. S3c, d), and absent in secondary recipients (Fig. 1g, i), demonstrating that L-biased or L-restricted blood contribution reflects the prolonged longevity of lymphocytes compared to short-lived myeloid lineages,^7^ rather than a lymphopoietic bias of LT-HSCs. Of relevance to our findings, recent clonal tracking of native hematopoiesis suggested that myeloid and lymphoid cells are mainly derived from embryonic multipotent progenitors (eMPPs) independently of HSCs, at least in the first months after birth, and that eMPPs remain the principal source of lifelong lymphoid contribution.^8^ In our studies, many pnHSCs, enriched among *Vwf*-tdTomato^−^ LSKCD150^+^CD48^−^ cells, gave LT robust reconstitution primarily of lymphocytes, and further analysis of reconstituted BM demonstrated that these L-biased/restricted cells were not true LT-HSCs, and might therefore be more closely related to short-term (ST) HSCs or eMPPs.

*Vwf*-tdTomato expression in pnHSCs was 59% and 47% in liver and BM, respectively (Supplementary information, Fig. S4a), comparable to adult BM.^2,4^ Based on index-sort data, the frequency of single reconstituting *Vwf*-tdTomato^+^ and *Vwf*-tdTomato^−^ pnHSCs was comparable (Supplementary information, Fig. S4b). Although P- and PEM-bias was rarer than in adult HSCs, these properties were exclusive to *Vwf*-tdTomato^+^ pnHSCs (Fig. 1j, k). No significant differences were observed between liver and BM for lineage bias/restriction of *Vwf*-tdTomato^+^ and *Vwf*-tdTomato^−^ pnHSCs (Supplementary information, Fig. S4c, d). This demonstrates that, although much less prominent than for adult *Vwf*-reporter^+^ HSCs, the process of P- and PEM-bias/restriction has initiated in *Vwf*-reporter^+^ HSCs at the time of birth. Moreover, single multilineage *Vwf*-tdTomato^−^ pnHSCs did not robustly replenish *Vwf*-tdTomato^+^ BM LSKCD150^+^CD48^−^ cells in primary recipients (Supplementary information, Fig. S4e, f). Together with all ED14.5 fetal liver HSCs being *Vwf*-reporter^+^,^4^ this suggests that the hierarchical relationship between *Vwf*-reporter^+^ and *Vwf*-reporter^−^ adult HSCs,^2,4^ with *Vwf*-reporter^+^ HSCs giving rise to *Vwf*-reporter^−^ HSCs but not vice versa, is already established in pnHSCs, although this should be further validated through in vivo functional HSC assays as previously reported for adult *Vwf*-reporter^+^ and *Vwf*-reporter^−^ HSCs.^4^

We next compared the transcriptional landscapes of *Vwf*-tdTomato^+^ and *Vwf*-tdTomato^−^ LSKCD150^+^CD48^−^ liver pnHSCs through single-cell RNA sequencing.^9^ Dimensionality reduction analysis did not reveal distinct clustering associated with *Vwf* expression (Supplementary information, Figs. S5, S6a, b), but 32 differentially expressed genes (DEGs) were identified between *Vwf*-tdTomato^−^and *Vwf*-tdTomato^+^ pnHSCs (Supplementary information, Fig. S6c and Table S2), with several genes correlating with *Vwf*-tdTomato level (Fig. 1l). We therefore also compared *Vwf*-tdTomato^−^ and *Vwf*-tdTomato^high^ pnHSCs, revealing 162 DEGs with overall higher fold changes (Fig. 1m; Supplementary Table S2) that more clearly separated pnHSC subclusters (Supplementary information, Fig. S6d, e). DEGs more highly expressed by *Vwf*-tdTomato^+/high^ pnHSCs included the maternally imprinted long-noncoding RNAs *H19*, *Meg3*, and *Rian* (Fig. 1n), located in the *H19*–*Igf2* and *Dlk1*–*Dio3* loci demonstrated to be selectively highly expressed in LT-HSCs and critical for establishment and maintenance of embryonic and adult HSCs.^10,11^ *Igf2* and its downstream target *Cdkn1c* are also important for maintaining HSC quiescence (Fig. 1n).^12^ These genes are located within distinct loci on mouse chromosome 7 and 11, implicating shared epigenetic regulation promoting higher expression in *Vwf*-tdTomato^+/high^ pnHSCs. DEGs also included genes associated with lineage-biased adult HSCs (Supplementary information, Fig. S6f) contributing to enrichment in *Vwf*-tdTomato^+/high^ pnHSCs of signatures associated with LT-HSCs,^13^ serial engraftment,^3^ and megakaryocyte (Mk) lineage bias^3^ (Fig. 1o). In support of L-biased *Vwf*-tdTomato^−^ LSKCD150^+^CD48^−^ perinatal cells being enriched in HSPCs closely related to eMPPs, *Flt3*, a hallmark gene expressed by eMPPs,^8^ was one of the most upregulated genes in *Vwf*-tdTomato^–^ when compared to *Vwf*-tdTomato^+/high^ LSKCD150^+^CD48^–^ cells (Fig. 1l, m; Supplementary information, Fig. S6c–f).

Our single HSC transplantations were performed under hematopoietic stress conditions using myelo-ablated recipients, therefore mimicking clinical BM transplantation rather than steady-state hematopoiesis, although, as in corresponding studies of adult HSCs,^2^ lineage-bias remained quite stable many months after transplantation when hematopoiesis had become more normalized. Regardless, the purpose of single HSC transplantations is to perform definitive clonal tracking of blood lineage contribution over time by a single cell with extensive LT self-renewal capacity, and our kinetic fate-mapping of post-transplantation outputs from single pnHSCs provides new insights into the blood replenishment potentials of definitive HSCs at birth, as compared to mice transplanted with adult single HSCs.^2^ Whereas a large fraction of adult HSCs replenishes exclusively PEM lineages and frequently are P-biased, these lineage restrictions and biases are rare or absent in pnHSCs. Unlike previous studies,^6^ we did not find that M-bias increased in HSCs residing in BM at late gestation, as no significant differences were observed in lineage-biases in liver and BM. Our functional data are congruent with published molecular data^14^ demonstrating that liver and BM pnHSCs are transcriptionally similar, and rather cluster by developmental stage.

Our study does not address the earliest time point of P-bias/restriction emergence, although the low frequency of P- and PEM-bias/restriction observed in pnHSCs shows that this property, shared by a large fraction of adult HSCs, emerges primarily after birth. While our single-cell functional and transcriptional data show that the extent of P- and PEM-bias/restriction is very limited in pnHSCs when compared to adult HSCs, in agreement with previous molecular analysis of Mk priming,^15^ we nevertheless observed that this process had initiated at birth in a pattern reminiscent of adult HSCs. Indeed, the observed lineage biases (P, PEM, and PEMB) were identical to those of adult HSCs, and P- and PEM-biases were exclusively a property of pnHSCs expressing *Vwf-*tdTomato, a hallmark of P- and PEM-restricted HSCs in adult BM.^2^

To what degree the distinct lineage-biases of HSCs, including P- and PEM-bias, are intrinsically and/or extrinsically determined remains to be explored further. However, the fact that both adult^2–4^ and perinatal LT-HSCs show similar lineage-biases upon transplantation into primary and secondary recipients, as well as transcriptional lineage priming corresponding to their lineage-bias upon transplantation, suggests that this is at least in part an intrinsically programmed and stable HSC property. The involvement of epigenetic programming in the P-bias of HSCs^16^ is also compatible with an interplay between intrinsic and extrinsic cues.

### Supplementary information

Supplementary information, Materials and Methods

Supplementary information, Table S1

Supplementary information, Table S2

Supplementary information, Fig. S1

Supplementary information, Fig. S2

Supplementary information, Fig. S3

Supplementary information, Fig. S4

Supplementary information, Fig. S5

Supplementary information, Fig. S6

## Data Availability

Sequencing data have been deposited at ArrayExpress under the following accession number: E-MTAB-13293.
