# Supplementary material for: Platelet and myeloid lineage biases of transplanted single perinatal mouse hematopoietic stem cells
Source: Cell Res. 2023 Sep 6;33(11):883–6. doi: 10.1038/s41422-023-00866-4 (PMC10624660; doi:10.1038/s41422-023-00866-4)
Supplement: Supplementary file 1 — Supplementary information, Materials and Methods [file 41422_2023_866_MOESM1_ESM.pdf]

SUPPLEMENTARY INFORMATION

Materials and Methods

Mice

Mouse experiments were performed in accordance with the regulations of the Swedish Board of Agriculture and the UK Home Office. Male and female B6.SJL-Ptprc^{a} Pepc^{b}/BoyJ and B6.SJL-Ptprc^{a} Pepc^{b}/BoyCrl (CD45.1) mice, 7-18 weeks old, were used as recipients in all transplantations, and as donors of unfractionated bone marrow (BM) competitor cells in primary transplantations. Donor cells were harvested for transplantation from *Vwf*-tdTomato$^{tg/+}$;*Gata1*-eGFP$^{tg/+}$ mice[1-3] and for single cell RNA sequencing from *Vwf*-tdTomato$^{tg/+}$ mice. In these mouse lines, generated by bacterial artificial chromosome recombinant engineering, transcription from the promoters of the *Vwf* and *Gata1* genes is reported by fluorescent proteins. Timed matings were set up between *Vwf*-tdTomato$^{tg/+}$;*Gata1*-eGFP$^{tg/+}$, *Vwf*-tdTomato$^{tg/+}$, or *Gata1*-eGFP$^{tg/+}$ males, and wildtype C57BL/6, *Gata1*-eGFP$^{tg/+}$, or *Vwf*-tdTomato$^{tg/+}$ females, respectively. The morning after mating was defined as embryonic day (ED) 0.5. We then isolated single perinatal hematopoietic stem cells (pnHSCs) from *Vwf*-tdTomato$^{tg/+}$;*Gata1*-eGFP$^{tg/+}$ or *Vwf*-tdTomato$^{tg/+}$ CD45.2 mice[3] directly (< 24 hours) after birth (postnatal day zero, PD0), which occurred shortly before ED19.5, as previously shown for C57BL/6 mice.[4] Individual neonates were phenotyped by flow cytometry analysis of tdTomato and eGFP in blood, and cells from 1-4 tissues of identical phenotype were pooled for pnHSC sorting for transplantation (≥1 litter per sort sample) or for RNA sequencing (single litter per sort sample).

Single pnHSC transplantations

Single cell transplantations were performed as previously described.[3] Briefly, pelvic and leg bones or liver were crushed and filtered in phosphate buffered saline (PBS) supplemented with 10% fetal calf serum (FCS, Sigma-Aldrich; Darmstadt, Germany) and 2 mM EDTA (Sigma-Aldrich). Antibody staining (Supplementary information, Table S1) was performed in the same media for 15-20 minutes at 4°C, after pre-incubation with purified CD16/CD32 antibody to block Fc receptors. Single ED19.5/PD0 liver and BM CD45.2^{+}Lineage^{-}(Ter119, Gr1, B220, CD8a, CD5)Sca1^{+}Kit^{+}(LSK)CD150^{+}CD48^{-} pnHSCs were index-sorted with 100 µm nozzle into 96-well round-bottom tissue culture plates (Corning; Corning, New York, USA) using FACSAriaII or FACSAria Fusion cell sorters (BD Biosciences; Franklin Lakes, New Jersey, USA) with an automated cell deposition unit. Deposition of single fluorescent beads was assessed before and after pnHSC sorts to confirm sorting of a single particle into each well. Each single pnHSC was sorted into a well with IMDM (Gibco - Thermo Fisher Scientific; Waltham, Massachusetts, USA), 20% BIT9500 (STEMCELL Technologies; Vancouver, Canada), penicillin/streptomycin (Gibco - Thermo Fisher Scientific) at 100U/mL and 0.1mg/mL, respectively, 2 mM L-glutamine (Sigma-Aldrich), and 0.1 mM 2-mercaptoethanol (Sigma-Aldrich), then mixed with 2.5×10^{5} CD45.1 unfractionated adult BM competitor cells in the same media, and injected in the lateral tail vein of lethally irradiated CD45.1 mice (9.6-11 Gy split into two equal doses 4 hours apart; X-ray or Cesium-137 source). Seven sorts were performed for a total of 252 single pnHSC transplanted mice (23-42 recipients per experiment), and single sorted pnHSCs were distributed randomly within each cohort of recipients regardless of tissue of origin or index-sort information. Index-sort information was used to analyse reconstitution data of *Vwf*-tdTomato^{+} and *Vwf*-tdTomato^{-} fractions for 54 of the 55 long-term (LT) reconstituting pnHSCs; index data were not appropriately recorded for 1 pnHSC.

Reconstitution analysis

Reconstitution of peripheral blood (PB) lineages (platelet [P], erythroid [E], myeloid [M], B cell [B] and T cell [T]), BM hematopoietic stem and progenitor cells (HSPCs), and double-positive (DP) thymic progenitors was analyzed by flow cytometry. Lineage-biased and lineage-restricted PB reconstitution patterns were categorized as described below and as in previous studies.[3] PB was collected from the lateral tail vein into lithium heparin microvettes (Sarstedt; Nümbrecht, Germany). Platelets were collected by PB centrifugation (100*g*, 10 minutes, room temperature) and mixed with small amount of red blood cell precipitate for the platelet/erythroid panel. For the leukocyte panel, supernatant was collected after 1:1 incubation (20-30 minutes, 37°C) in a solution of 2% weight/volume Dextran-500 (Sigma-Aldrich) and remaining erythrocytes were lysed (2 minutes, room temperature) with 0.8% ammonium chloride solution (STEMCELL Technologies). Unfractionated BM cells were enriched with Kit (CD117) MicroBeads (Miltenyi Biotec; Bergisch Gladbach, Germany) according to manufacturer instructions. Cells were pre-incubated with purified anti-CD16/CD32 before staining, except in the case of platelets/erythroid cells (no blocking) and BM myeloid progenitors (pre-incubation with fluorophore-conjugated anti-CD16/CD32). Antibody staining (Supplementary information, Table S1) was performed for 15-20 minutes at 4°C in PBS with 1-5% FCS and 2 mM EDTA. Samples were recorded with LSRII and LSR Fortessa cytometers (BD Biosciences) and analyzed with FlowJo software (BD Biosciences).

Peripheral blood reconstitution parameters

PB lineages were analyzed with two separate staining panels (Supplementary information, Table S1) and phenotypically defined as follows. HSC (donor)-derived platelets: $Ter119^{-}CD150^{+}CD41^{+}$*Vwf*-$tdTomato^{+}$*Gata1*-$eGFP^{+}$. Donor-derived erythroid cells: $Ter119^{+}$ $CD150^{-}CD41^{-}$*Vwf*-$tdTomato^{-}$*Gata1*-$eGFP^{+}$. Myeloid cells: $NK1.1^{-}CD41^{-}CD4/CD8a^{-}CD19^{-}$ $CD11b^{+}$. B cells: $NK1.1^{-}CD41^{-}CD4/CD8a^{-}CD11b^{-}CD19^{+}$. T cells: $NK1.1^{-}CD41^{-}CD11b^{-}$ $CD19^{-}CD4/CD8a^{+}$. Donor-derived leukocytes (M, B and T cells) were distinguished from recipient/competitor leukocytes based on expression of CD45.2 and CD45.1, respectively. An anti-CD41 antibody was included in PB analysis for exclusion of leukocytes with attached platelets. PB reconstitution of primary recipients was analyzed at 4-5, 8-9, 16-18, and 25-26 weeks post-transplantation. Additional PB analysis was performed at 32-55 weeks in five cohorts. Recipients were considered reconstituted by a potential LT-HSC when its contribution was ≥0.1% to at least one PB lineage at 16-26 weeks post transplantation. The threshold of 0.1% was set as low to allow a direct comparison between pnHSC data and our previously published corresponding data on the transplantation of adult BM single HSCs,[1,3] and also since stable low reconstitution (0.1% to 1%) of PB myeloid cells and/or platelets ≥16 weeks post-transplantation might reflect the activity of true LT-HSCs. PB reconstitution was thus used to identify a broad range of potential LT-HSCs, which we then further investigated through analysis of primary recipients and through secondary transplantation.

Reconstitution data from primary recipients that did not survive until PB readout at 25-26 weeks post-transplantation were excluded. Donor contribution to a PB lineage was considered undetectable if <0.01%, and primary recipients with <0.01% reconstitution of all lineages at ≥8 weeks post-transplantation were not analyzed further. The threshold of 0.01% was defined as previously described,[3] requiring ≥10,000 flow cytometry events recorded for each PB lineage and ≥5 donor-derived events for a reconstitution of >0.01% to be considered reliable. Lineage-restricted reconstitution patterns were defined based on undetectable (<0.01%) contribution of the single transplanted HSC to one or more PB lineages. Lineage bias was defined as HSC percentage contribution to one or some (but not all) PB lineages, that was at least threefold (3×) higher than the percentage contribution to the remaining lineages.

Lymphoid bias (L-bias): B and/or T cell reconstitution levels ≥3× higher than the reconstitution of both P and M lineages. PEMB-bias: reconstitution levels of P, E, M, and B cell lineages all ≥3× higher than the reconstitution of the T cell lineage and with <3× difference between P, E, M, and B cells. PEM-bias: reconstitution levels of P, E, and M lineages all ≥3× higher than the reconstitution of the B and T cell lineages and with <3× difference between P, E, and M lineages. P-bias: reconstitution level of P lineage >3× higher than the reconstitution of the E, M, B, and T cell lineages. Patterns in which no single lineage or combination of lineages showed reconstitution levels ≥3× higher than all other lineages were considered not to be lineage biased.

Bone marrow and thymus stem-progenitor reconstitution analysis

HSPCs were analyzed by flow cytometry in primary recipients at 26-55 weeks post-transplantation and PB lineage analysis was repeated at this terminal time point unless performed in the previous two weeks. The following HSPC populations were analyzed based on previous studies:[3,5-7]

Long-term HSC (LT-HSC): Lineage^{-}Sca1^{+}Kit^{+} (LSK) Flt3^{-}CD150^{+}CD48^{-}

Short-term HSC (ST-HSC): LSKFlt3^{-}CD150^{-}CD48^{-}

Multipotent progenitor 2 (MPP2): LSKFlt3^{-}CD150^{+}CD48^{+}

MPP3: LSKFlt3^{-}CD150^{-}CD48^{+}

MPP4: LSKFlt3^{+}

Megakaryocyte progenitor (MkP): Lineage^{-}Sca1^{-}Kit^{+} (LK) CD150^{+}CD41^{+}

Pre-megakaryocyte/erythroid progenitor (PreMegE): LKCD41^{-}CD16/32^{-}CD150^{+}CD105^{-}

Colony forming unit-erythroid (CFU-E): LKCD41^{-}CD16/32^{-}CD150^{-}CD105^{+}

Pre-granulocyte/monocyte progenitor (PreGM): LKCD41^{-}CD16/32^{-}CD150^{-}CD105^{-}

Granulocyte/monocyte progenitor (GMP): LKCD41^{-}CD150^{-}CD16/32^{+}

Pro-B cells: Lineage^{-}B220^{low}CD19^{+}Kit^{+}IgM^{-}

Thymic double positive (DP) T cell progenitors: Lineage^{-}CD4^{+}CD8a^{+}

Lineage cocktail for LSK and LK panels consisted of Ter119, CD11b, Gr1, B220, CD4, CD8a, and CD5; for Pro-B cells it was Ter119, Gr1, F4/80, CD3e, NK1.1, and CD11c; and for DP thymocytes it was Ter119, Gr1, CD11b, NK1.1, CD11c, B220, and CD19.

Single HSC contribution to HSPCs was calculated as the percentage of CD45.2^{+} cells in each population, except for CFU-E where percentage *Gata1*-eGFP^{+} cells were quantified instead due to low CD45 expression in erythroid progenitors.[2] The reconstitution of a HSPC population was only considered to be positive and reliable if ≥0.01% and with ≥5 donor-derived events recorded.

Secondary transplantations

Secondary transplantations were performed at the time of HSPC reconstitution analysis of primary recipients, 28-55 weeks after the single HSC transplantation. 10-20 × 10^6 unfractionated BM cells from primary recipients were transplanted in PBS with 1% FCS by lateral tail vein injection into lethally irradiated secondary CD45.1 recipients (10-11 Gy split into two equal doses 4 hours apart; X-ray or Cesium-137 source). Cells from each primary donor were transplanted into 1-3 secondary recipients.

PB of secondary recipients was analyzed at 16-18 weeks post-transplantation or within two weeks of terminal HSPC reconstitution analysis after secondary transplantation. Secondary recipients with ≥0.1% reconstitution in at least one PB lineage at 16-18 weeks post-transplantation were considered to be LT reconstituted. BM HSPCs and thymic progenitors were only analyzed in secondary recipients with LT PB reconstitution. Data from secondary recipients that did not survive until the PB readout were excluded.

For each group of primary or secondary recipients, the mean reconstitution percentage of each HSPC and PB population was calculated by including all recipients with or without detectable reconstitution of that particular population. For HSPC and PB populations with no detectable donor contribution in any recipients, the mean detection threshold of all recipients was used as the overall threshold. In all mean calculations, reconstitution values <0.01% were set at 0.01% (level of detection).

Single cell RNA sequencing

Single CD45.2^{+}LSKCD150^{+}CD48^{-} pnHSCs were index-sorted, as described above, from *Vwf*-tdTomato^{+} ED19.5/PD0 livers (3 samples, each sample representing a separate litter and consisting of 1-3 livers). Only female donors were used, selected based on sex-specific genotyping using simplex PCR targeting the *Rbm31x/y* genes as previously described,[8] and expression of *Vwf*-tdTomato reporter was confirmed by flow cytometry before sample pooling. As an internal batch control, each plate contained one row of single HSCs (LSKCD150^{+}CD48^{-}, 24 cells/plate) sorted from the same frozen BM sample of a 4-week old wild-type mouse.
Smart-seq3xpress libraries were prepared as previously described,[9] with some modifications. Briefly, cells were sorted into 384 well plates with each well containing 3μL Vapor-Lock (Qiagen, Hilden, Germany) and 0.3μL lysis buffer adjusted to reverse transcription (RT) volume: 0.125μM OligodT30VN (5'-Biotin-ACGAGCATCAGCAGCATACGAT$_{30}$VN-3'; IDT), 0.5mM dNTPs/each, 0.1% Triton X-100, 5% PEG8000, 0.4μL Rnase Inhibitor 40 U μL^{-1} (Takara Bio, San Jose, California, USA). The collection plates were briefly centrifuged after cell sorting and stored at -80°C until further processing. Before RT, plates were denatured at 72°C for 10 minutes, followed by addition of 0.1μL of RT mix: 25mM Tris-HCL pH 8.4 (Thermo Fisher Scientific), 30mM NaCl (Ambion - Thermo Fisher Scientific), 1mM GTP (Thermo Fisher Scientific), 2.5mM MgCl2 (Ambion - Thermo Fisher Scientific), 8mM DTT (Thermo Fisher Scientific), 0.25 U μl^{-1} Rnase Inhibitor (Takara Bio), 0.75μM Template Switching Oligo (TSO) (5′-Biotin-AGAGACAGATTGCGCAATGNNNNNNNNWWrGrGrG-3′; IDT), 2 U μl^{-1} of Maxima H Minus reverse transcriptase (Thermo Fisher Scientific). Plates were briefly centrifuged after dispensing to ensure merging of lysis and RT volumes underneath the Vapor-Lock overlay, then incubated at 42°C for 90 minutes, followed by 10 cycles of 50°C for 2 minutes and 42°C for 2 minutes. After RT, 0.6μL PCR mix was dispensed to each well: 1× SeqAmp PCR buffer (Takara Bio), 0.025 U μl^{-1} of SeqAmp polymerase (Takara Bio), 0.5 μM Smartseq3 forward primer (5′-TCGTCGGCAGCGTCAGATGTGTATAAGAGACAGATTGCGCAATG-3′; IDT), 0.5 μM Smartseq3 reverse primer (5′-ACGAGCATCAGCAGCATACGA-3′; IDT). Plates were briefly centrifuged and subjected to pre-amplification PCR: 1 minute at 95°C for initial denaturation; 14 cycles consisting of 10 seconds at 98°C, 30 seconds at 65°C, 4 minutes at 68°C; 10 minutes at 72°C for final elongation. Pre-amplified libraries were then diluted with 9μL H_2O and 1μL of diluted cDNA was transferred from each well into a new 384 well plate where Tagmentation was performed by adding 1μL of tagmentation mix per well: 1x tagmentation buffer (10 mM Tris pH 7.5, 5 mM $MgCl_2$, 5% DMF), 0.003μL Tagmentation DNA Enzyme 1 (TDE1; Illumina DNA sample preparation kit, Illumina, San Diego, California, USA). Plates were incubated for 10 minutes at 55°C and the reaction was stopped by the addition of 0.5μL 0.2% SDS to each well. Index PCR was carried out after the addition of 3.5μL custom Nextera Index primers (0.5μM, Illumina) by dispensing 2μL of PCR mix: 1× Phusion Buffer (Thermo Fisher Scientific), 0.01 U μl^{-1} of Phusion DNA polymerase (Thermo Fisher Scientific), 0.025% Tween-20, 0.2 mM dNTP each. Index PCR: 3 minutes at 72°C; 30 seconds at 95°C; 12 cycles consisting of 10 seconds at 95°C, 30 seconds at 55°C, 1 minute at 72°C; 5 minutes at 72°C. Each indexed library plate was pooled by gently pulsing the

centrifuge to <200*g* while using a 300mL robotic reservoir (Nalgene - Thermo Fisher Scientific) fitted with a custom 3D-printed scaffold. The pooled libraries were then purified with homemade 22% PEG beads at a ratio of 1 sample to 0.7 beads.
Smart-seq3xpress libraries were sequenced on a MGI DNBSEQ G400RS platform (MGI, Shenzhen, China). Prior to sequencing, single stranded circular (ssCir) DNA libraries were generated using the MGIEasy Universal Library Conversion Kit (MGI). Adapter conversion PCR was carried out for 5 cycles on 50ng of final pooled library, following circularization of 1pmol dsDNA according to manufacturer's protocol. DNA nanoballs (DNBs) were created from 60 fmol of ssCir DNA library pools using a custom rolling-circle amplification primer (5′-TCGCCGTATCATTCAAGCAGAAGACG-3′, IDT). DNBs were sequenced paired-end 100 bp (PE100) using the following custom sequencing primers:
Read 1: 5′-TCGTCGGCAGCGTCAGATGTGTATAAGAGACAG-3′
MDA: 5′-CGTATGCCGTCTTCTGCTTGAATGATACGGCGAC-3′
Read 2: 5′-GTCTCGTGGGCTCGGAGATGTGTATAAGAGACAG-3′
i7 index: 5′-CCGTATCATTCAAGCAGAAGACGGCATACGAGAT-3′
i5 index: 5′-CTGTCTCTTATACACATCTGACGCTGCCGACGA-3′

Analysis of single cell RNA sequencing data
Raw FASTQ files were preprocessed with the zUMIs pipeline (2.9.7).[10,11] Reads containing unique molecular identifier (UMI) were identified by the (ATTGCGCAATG) pattern, allowing up to two mismatches, and reads were filtered for low quality UMIs (3 bases < phred 20) and index barcodes (4 bases < phred 20). Mapping to the mouse genome (mm10) was then performed with STAR (2.7.3).[12] Read counts and UMI counts were calculated using Ensembl gene annotations (GRCm38.91).[13]
Subsequent analysis was performed in R (4.1.2) using Seurat (4.0.5).[14] Cells with >1,000 genes detected and genes detected in ≥50 cells were selected for downstream analysis. After quality control filtering, internal control cells (n=128) and pnHSCs lacking index-sort information (n=22) were removed. Index-sort information was then used to identify *Vwf*-tdTomato^{-} (n=1128), total *Vwf*-tdTomato^{+} (n=572), *Vwf*-tdTomato^{low} (50% lowest Tomato expressing within *Vwf*-tdTomato^{+} cells; n=289), and *Vwf*-tdTomato^{high} (50% highest Tomato expressing within *Vwf*-tdTomato^{+} cells; n=283) fractions among the final 1700 cells. *Vwf*-tdTomato^{-} and *Vwf*-tdTomato^{+} gating (Supplementary information, Fig. S4a) was aided by analysis of HSCs lacking expression of the tdTomato transgene, and the *Vwf*-tdTomato^{+} gate used for each donor sample was split into two equal halves defined as *Vwf*-tdTomato^{low} and *Vwf*-tdTomato^{high}. Counts were normalised, sample effect was regressed out, and variable genes were selected using the sctransform package (0.3.2).[15,16] Dimensionality reduction was performed using principal component analysis. Uniform manifold approximation and projection (UMAP) was performed on the first five principle components, determined by inspection of the elbow plot, with default parameters. Based on analysis of internal control cells among the different plates (n=128 cells), no batch effect between the individual plates was observed. Gene signature scores were computed with AUCell package (1.16.0)[17] using previously published gene sets for LT-HSC ("molecular overlap", MolO),[18] serial engrafter HSC,[19] and Mk-biased HSC.[19] Differential gene expression analysis and hierarchical clustering heatmap were performed with the pheatmap package (1.0.12), and clusters were sorted with dendsort (0.3.4). Differential gene expression between *Vwf*-tdTomato^{-}, total *Vwf*-tdTomato^{+}, *Vwf*-tdTomato^{low}, and *Vwf*-tdTomato^{high} HSCs was analysed using the sctransform normalized expression values using the Wilcoxon rank-sum test to test for difference in expression magnitude combined with Fisher's exact test to test for difference in expression frequency.[20] Genes with log1p fold change >0.2 or <–0.2 and with combined p-value <0.05 were considered to be differentially expressed genes (DEGs; Supplementary information, Table S2). AUCell

scores between *Vwf*-tdTomato^{-}, total *Vwf*-tdTomato^{+}, and *Vwf*-tdTomato^{high} HSCs were tested using Wilcoxon rank-sum test. Spearman rank correlation between gene expression and level of *Vwf*-tdTomato reporter was computed with the cor.test function in R.

Statistical analysis
Statistical significance of the frequency of reconstituted mice, the distribution of blood reconstitution patterns, and the distribution of overall lineage biases between groups was evaluated with two-tailed Fisher's exact test using R (4.1.2). P-values <0.05 were considered statistically significant.

REFERENCES

1 Sanjuan-Pla, A. *et al.* Platelet-biased stem cells reside at the apex of the haematopoietic stem-cell hierarchy. *Nature* **502**, 232-236 (2013). https://doi.org:10.1038/nature12495

2 Drissen, R. *et al.* Distinct myeloid progenitor-differentiation pathways identified through single-cell RNA sequencing. *Nature immunology* **17**, 666-676 (2016). https://doi.org:10.1038/ni.3412

3 Carrelha, J. *et al.* Hierarchically related lineage-restricted fates of multipotent haematopoietic stem cells. *Nature* **554**, 106-111 (2018). https://doi.org:10.1038/nature25455

4 Murray, S. A. *et al.* Mouse gestation length is genetically determined. *PloS one* **5**, e12418 (2010). https://doi.org:10.1371/journal.pone.0012418

5 Kiel, M. J., Yilmaz, O. H., Iwashita, T., Terhorst, C. & Morrison, S. J. SLAM family receptors distinguish hematopoietic stem and progenitor cells and reveal endothelial niches for stem cells. *Cell* **121**, 1109-1121 (2005). https://doi.org:10.1016/j.cell.2005.05.026

6 Pronk, C. J. *et al.* Elucidation of the phenotypic, functional, and molecular topography of a myeloerythroid progenitor cell hierarchy. *Cell stem cell* **1**, 428-442 (2007). https://doi.org:10.1016/j.stem.2007.07.005

7 Pietras, E. M. *et al.* Functionally distinct subsets of lineage-biased multipotent progenitors control blood production in normal and regenerative conditions. *Cell stem cell* **17**, 35-46 (2015). https://doi.org:10.1016/j.stem.2015.05.003

8 Tunster, S. J. Genetic sex determination of mice by simplex PCR. *Biol Sex Differ* **8**, 31 (2017). https://doi.org:10.1186/s13293-017-0154-6

9 Hagemann-Jensen, M., Ziegenhain, C. & Sandberg, R. Scalable single-cell RNA sequencing from full transcripts with Smart-seq3xpress. *Nature biotechnology* **40**, 1452-1457 (2022). https://doi.org:10.1038/s41587-022-01311-4

10 Parekh, S., Ziegenhain, C., Vieth, B., Enard, W. & Hellmann, I. zUMIs - A fast and flexible pipeline to process RNA sequencing data with UMIs. *Gigascience* **7** (2018). https://doi.org:10.1093/gigascience/giy059

11 Hagemann-Jensen, M. *et al.* Single-cell RNA counting at allele and isoform resolution using Smart-seq3. *Nature biotechnology* **38**, 708-714 (2020). https://doi.org:10.1038/s41587-020-0497-0

12 Dobin, A. *et al.* STAR: ultrafast universal RNA-seq aligner. *Bioinformatics* **29**, 15-21 (2013). https://doi.org:10.1093/bioinformatics/bts635

13 Martin, F. J. *et al.* Ensembl 2023. *Nucleic acids research* **51**, D933-D941 (2023). https://doi.org:10.1093/nar/gkac958

14 Hao, Y. *et al.* Integrated analysis of multimodal single-cell data. *Cell* **184**, 3573-3587 e3529 (2021). https://doi.org:10.1016/j.cell.2021.04.048

15 Hafemeister, C. & Satija, R. Normalization and variance stabilization of single-cell RNA-seq data using regularized negative binomial regression. *Genome Biol* **20**, 296 (2019). https://doi.org:10.1186/s13059-019-1874-1

16 Choudhary, S. & Satija, R. Comparison and evaluation of statistical error models for scRNA-seq. *Genome Biol* **23**, 27 (2022). https://doi.org:10.1186/s13059-021-02584-9

17 Aibar, S. *et al.* SCENIC: single-cell regulatory network inference and clustering. *Nature methods* **14**, 1083-1086 (2017). https://doi.org:10.1038/nmeth.4463

18 Wilson, N. K. *et al.* Combined single-cell functional and gene expression analysis resolves heterogeneity within stem cell populations. *Cell stem cell* **16**, 712-724 (2015). https://doi.org:10.1016/j.stem.2015.04.004

19 Rodriguez-Fraticelli, A. E. *et al.* Single-cell lineage tracing unveils a role for TCF15 in haematopoiesis. *Nature* **583**, 585-589 (2020). https://doi.org:10.1038/s41586-020-2503-6

20 Giustacchini, A. *et al.* Single-cell transcriptomics uncovers distinct molecular signatures of stem cells in chronic myeloid leukemia. *Nature medicine* **23**, 692-702 (2017). https://doi.org:10.1038/nm.4336
