# Supplementary material for: Platelet and myeloid lineage biases of transplanted single perinatal mouse hematopoietic stem cells
Source: Cell Res. 2023 Sep 6;33(11):883–6. doi: 10.1038/s41422-023-00866-4 (PMC10624660; doi:10.1038/s41422-023-00866-4)
Supplement: Supplementary file 2 — Supplementary information, Table S1 [file 41422_2023_866_MOESM2_ESM.pdf]

Supplementary information, Table S1: Antibodies and viability dyes used in flow cytometry analysis.

Marker	Conjugate	Clone	Supplier	Staining panels
B220 (CD45R)	BUV395	RA3-6B2	BioLegend	BM ProB
B220 (CD45R)	PE-Cy5	RA3-6B2	BioLegend	pnHSC sort<br>BM LK, LSK<br>Thymus
CD3e	PE-Cy5	145-2C11	BioLegend	BM ProB
CD4	APC-eF780	RM4-5	eBioscience	PB leukocytes
CD4	BV650	RM4-5	BioLegend	Thymus<br>PB leukocytes
CD4	PE-Cy5	RM4-5	BioLegend	BM LK, LSK
CD5	PE-Cy5	53-7.3	BioLegend	pnHSC sort<br>BM LK, LSK
CD8a	APC-eF780	53-6.7	eBioscience	Thymus<br>PB leukocytes
CD8a	PE-Cy5	53-6.7	BioLegend	pnHSC sort<br>BM LK, LSK
CD11b (Mac-1)	APC	M1/70	BioLegend	PB leukocytes
CD11b (Mac-1)	PE-Cy5	M1/70	BioLegend	BM LK, LSK<br>Thymus
CD11b (Mac-1)	PE-Cy7	M1/70	BioLegend	PB leukocytes
CD11c	PE-Cy5	N418	BioLegend	BM ProB<br>Thymus
CD16/CD32 (FcγIII/IIR)	APC	93	eBioscience	BM LK
CD16/CD32 (FcγIII/IIR)	PE-Cy7	93	eBioscience	BM LK
CD19	BV786	1D3	BD	BM ProB
CD19	eF450	1D3	eBioscience	BM ProB<br>PB leukocytes
CD19	PE-Cy5	1D3	eBioscience	Thymus<br>PB leukocytes
CD41	BV605	MWReg30	BD	BM LK
CD41	PE-Cy7	MWReg30	BioLegend	PB platelet/erythroid<br>PB leukocytes
CD45.1	BUV395	A20	BD	BM LK, LSK<br>Thymus<br>PB leukocytes
CD45.1	BUV737	A20	BD	BM ProB
CD45.1	BV605	A20	BioLegend	PB leukocytes
CD45.1	BV650	A20	BioLegend	BM ProB, LK, LSK
CD45.1	PE-Cy7	A20	BioLegend	Thymus<br>PB leukocytes
CD45.2	AF700	104	BioLegend	pnHSC sort<br>BM ProB, LK, LSK<br>Thymus
CD45.2	BUV737	104	BD	pnHSC sort

				BM LK, LSK<br>Thymus<br>PB leukocytes
CD48	APC	HM48-1	BioLegend	pnHSC sort<br>BM LSK
CD48	BUV 737	HM48-1	BD	BM LSK
CD105	BV421	MJ7/18	BD	BM LK
CD117 (cKit)	APC-eF780	2B8	eBioscience	pnHSC sort<br>BM LK, LSK
CD117 (cKit)	PE-Cy7	2B8	BioLegend	BM ProB
CD135 (Flt3)	BV421	A2F10	BioLegend	BM LSK
CD150 (SLAM)	APC	TC15-12F12.2	BioLegend	BM LK<br>PB platelet/erythroid
CD150 (SLAM)	BV785	TC15-12F12.2	BioLegend	pnHSC sort<br>BM LSK<br>PB platelet/erythroid
CD150 (SLAM)	PE-Cy7	TC15-12F12.2	BioLegend	pnHSC sort<br>BM LK, LSK
F4/80	PE-Cy5	BM8	BioLegend	BM ProB
Gr-1 (Ly-6G/C)	PE-Cy5	RB6-8C5	BioLegend	pnHSC sort<br>BM ProB, LK, LSK<br>Thymus
IgM	APC	Il/41	BD	BM ProB
NK1.1	Pacific Blue	PK136	BioLegend	Thymus<br>PB leukocytes
NK1.1	PE-Cy5	PK136	BioLegend	BM ProB<br>Thymus<br>PB leukocytes
Sca-1 (Ly-6A/E)	BV605	D7	BioLegend	pnHSC sort
Sca-1 (Ly-6A/E)	BV786	D7	BD	BM LK
Sca-1 (Ly-6A/E)	PE-Cy7	E13-161.7	BioLegend	pnHSC sort<br>BM LSK
Ter119 (Ly-76)	APC	TER-119	BioLegend	PB platelet/erythroid
Ter119 (Ly-76)	PE-Cy5	TER-119	BioLegend	pnHSC sort<br>BM ProB, LK, LSK<br>Thymus
Ter119 (Ly-76)	PerCP-Cy5.5	TER-119	eBioscience	PB platelet/erythroid
Viability dye:<br>7-aminoactinomycin D (7-AAD)			Sigma-Aldrich<br>Cayman Chemical Company	BM ProB, LK, LSK<br>PB leukocytes
Viability dye:<br>4',6-diamidino-2-phenylindole (DAPI)			Sigma-Aldrich	pnHSC sort<br>Thymus<br>PB leukocytes

Abbreviations: BM, bone marrow; ProB, Lineage^{-}B220^{low}CD19^{+}Kit^{+}IgM^{-} cells; pnHSC, perinatal hematopoietic stem cell; LK, Lineage^{-}Sca1^{-}Kit^{+} cells; LSK, Lineage^{-}Sca1^{+}Kit^{+} cells; PB, peripheral blood.
