# Supplementary material for: Platelet and myeloid lineage biases of transplanted single perinatal mouse hematopoietic stem cells
Source: Cell Res. 2023 Sep 6;33(11):883–6. doi: 10.1038/s41422-023-00866-4 (PMC10624660; doi:10.1038/s41422-023-00866-4)
Supplement: Supplementary file 4 — Supplementary information, Fig. S1 [file 41422_2023_866_MOESM4_ESM.pdf]

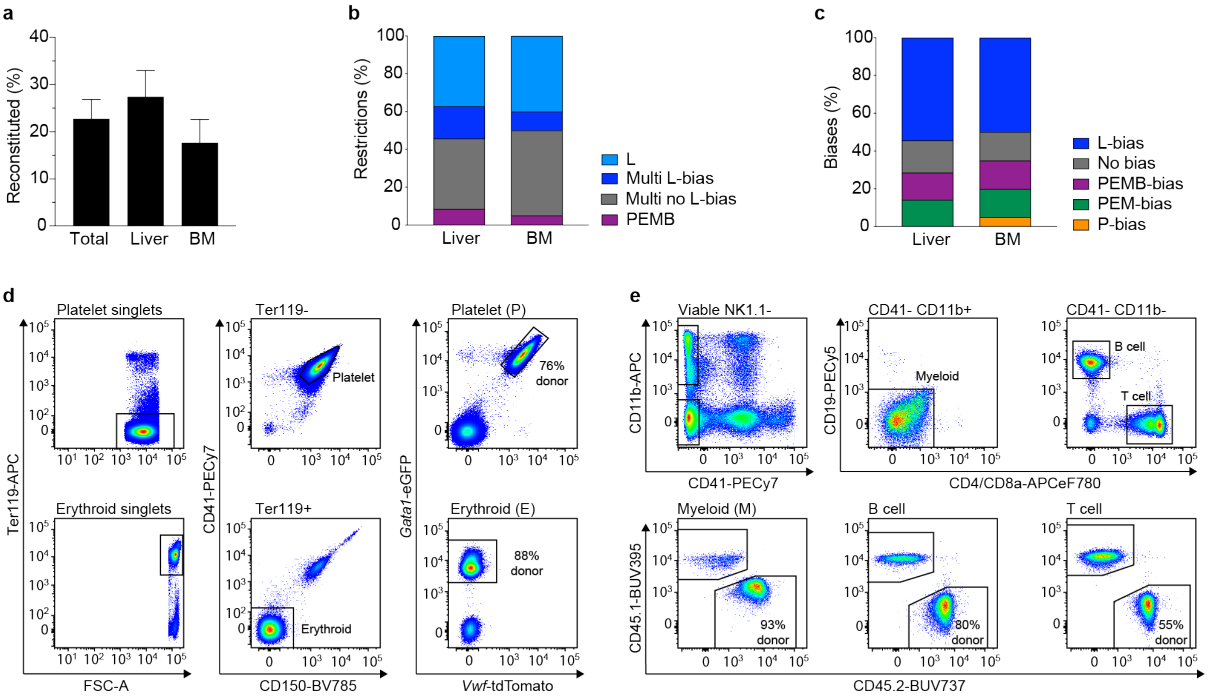

Supplementary information, Fig. S1: Comparison of lineage reconstitution patterns of single pnHSCs from liver and BM.

a Percentage (mean + SEM) of reconstituted mice (≥0.1% donor contribution to at least one PB lineage) at 25-26 weeks post-primary transplantation of single pnHSCs (liver, n=132; BM, n=120 recipients; 7 independent experiments). No statistically significant association between variables with Fisher's exact test (p=0.06753).

b-c Distribution of PB lineage restriction (**b**) and overall lineage bias (**c**) among reconstituted mice at 25-26 weeks post-primary transplantation (liver, n=35; BM, n=20). No statistically significant association between variables with Fisher's exact test (**b**, p=0.8977; **c**, p=0.8519).

d-e Representative flow cytometry reconstitution analysis of P and E (**d**), and M, B cell and T cell lineages (**e**) in PB of a primary recipient of a single pnHSC with long-term multilineage reconstitution without L bias. Percentage of donor-derived cells is shown for each population. Abbreviations: pnHSC, perinatal hematopoietic stem cell; BM, bone marrow; SEM, standard error of the mean; PB, peripheral blood; P, platelet; E, erythroid; M, myeloid; B, B cell; T, T cell; L, lymphoid (B and T cells).
