# Supplementary material for: Platelet and myeloid lineage biases of transplanted single perinatal mouse hematopoietic stem cells
Source: Cell Res. 2023 Sep 6;33(11):883–6. doi: 10.1038/s41422-023-00866-4 (PMC10624660; doi:10.1038/s41422-023-00866-4)
Supplement: Supplementary file 5 — Supplementary information, Fig. S2 [file 41422_2023_866_MOESM5_ESM.pdf]

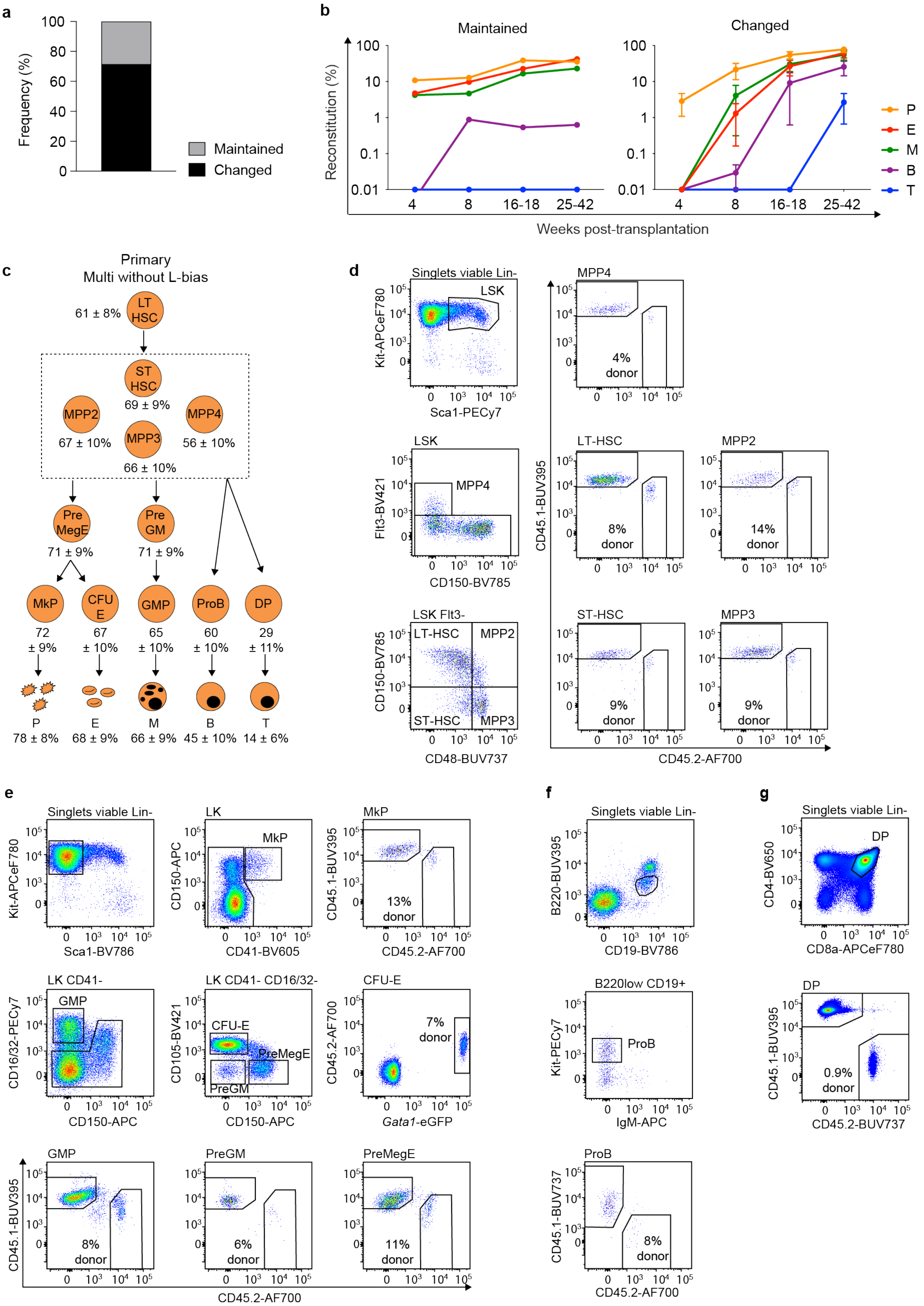

a
Frequency (%)
Maintained
Changed
b
Maintained
Changed
Reconstitution (%)
Weeks post-transplantation
P
E
M
B
T
c
Primary
Multi without L-bias
LT HSC
61 ± 8%
ST HSC
69 ± 9%
MPP2
67 ± 10%
MPP3
66 ± 10%
MPP4
56 ± 10%
Pre MegE
71 ± 9%
Pre GM
71 ± 9%
MkP
72 ± 9%
CFU E
67 ± 10%
GMP
65 ± 10%
ProB
60 ± 10%
DP
29 ± 11%
P 78 ± 8%
E 68 ± 9%
M 66 ± 9%
B 45 ± 10%
T 14 ± 6%
d
Singlets viable Lin-
LSK
Kit-APCeF780
Sca1-PECy7
MPP4
Flt3-BV421
CD150-BV785
LSK Flt3-
LT-HSC
MPP2
ST-HSC
MPP3
CD48-BUV737
4% donor
8% donor
14% donor
9% donor
9% donor
CD45.1-BUV395
CD45.2-AF700
e
Singlets viable Lin-
Kit-APCeF780
Sca1-BV786
LK
MkP
CD150-APC
CD41-BV605
13% donor
CD45.1-BUV395
CD45.2-AF700
LK CD41-
GMP
CD16/32-PECy7
CD150-APC
LK CD41- CD16/32-
CFU-E
PreMegE
PreGM
CD105-BV421
CD150-APC
7% donor
CD45.2-AF700
Gata1-eGFP
8% donor
6% donor
11% donor
f
Singlets viable Lin-
B220-BUV395
CD19-BV786
B220low CD19+
ProB
Kit-PECy7
IgM-APC
8% donor
CD45.1-BUV737
CD45.2-AF700
g
Singlets viable Lin-
DP
CD4-BV650
CD8a-APCeF780
0.9% donor
CD45.1-BUV395
CD45.2-BUV737

Supplementary information, Fig. S2: Multilineage contributions by single pnHSCs.
a Percentage of single pnHSC transplanted mice with PEMB-restricted PB reconstitution identified at 16-18 weeks post-primary transplantation, and that was maintained or changed to multilineage reconstitution at 25-42 weeks post-primary transplantation (n=7).
b PB reconstitution kinetics of maintained (n=2; mean) and changed (n=5; mean ± SEM) PEMB-restricted PB patterns.
c Mean (± SEM) contribution of transplanted single multilineage pnHSCs without L-bias to the hematopoietic stem and progenitor cell hierarchy of primary recipients (n=14, 27-49 weeks post-primary transplantation). Orange denotes contribution in all recipients.
d-f Representative flow cytometry reconstitution profiles of $Lineage^{-}Sca1^{+}Kit^{+}$ populations including LT-HSC, ST-HSC, multipotent progenitor fractions (MPP2, MPP3, MPP4) (**d**), $Lineage^{-}Sca1^{-}Kit^{+}$ progenitors including PreGM, GMP, PreMegE, MkP and CFU-E (**e**), and ProB cells (**f**) in Kit-enriched BM from a primary recipient of a single pnHSC with LT multilineage reconstitution without L-bias.
g Reconstitution analysis of thymic DP T cell progenitors in the same recipient. Percentage of donor-derived cells is shown for each population.
Abbreviations: pnHSC, perinatal hematopoietic stem cell; PEMB, platelet-erythroid-myeloid-B cell; PB, peripheral blood; SEM, standard error of the mean; L, lymphoid (B and T cells); LT-HSC, long-term HSC; ST-HSC, short-term HSC; MPP, multipotent progenitor; PreMegE, pre-megakaryocyte/erythroid progenitor; MkP, megakaryocyte progenitor; CFU-E, colony forming unit-erythroid; PreGM, pre-granulocyte/monocyte progenitor; GMP, granulocyte/monocyte progenitor; DP, double positive T cell progenitors.
