# Supplementary material for: Platelet and myeloid lineage biases of transplanted single perinatal mouse hematopoietic stem cells
Source: Cell Res. 2023 Sep 6;33(11):883–6. doi: 10.1038/s41422-023-00866-4 (PMC10624660; doi:10.1038/s41422-023-00866-4)
Supplement: Supplementary file 6 — Supplementary information, Fig. S3 [file 41422_2023_866_MOESM6_ESM.pdf]

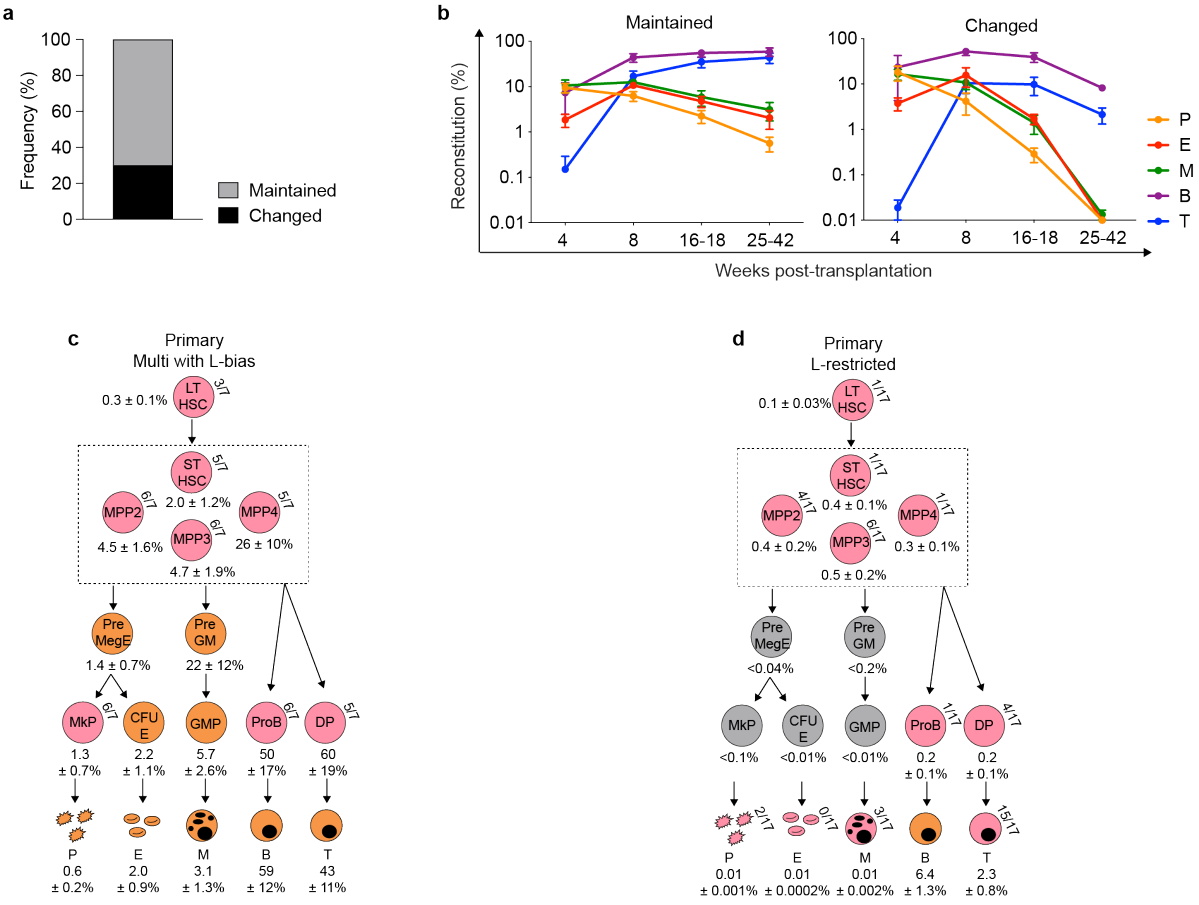

Supplementary information, Fig. S3: L-biased and L-restricted contributions by single pnHSCs.

a Percentage of single pnHSC transplanted mice with multilineage PB reconstitution with L-bias at 16-18 weeks post-primary transplantation that was maintained or changed to L-restricted reconstitution at 25-42 weeks post-primary transplantation (n=10).

b PB lineage reconstitution kinetics (mean ± SEM) of maintained (n=7) and changed (n=3) multilineage patterns with L-bias.

c-d Mean (± SEM) contribution of transplanted single multilineage pnHSCs with L-bias (**c**, n=7, 27-49 weeks post-primary transplantation) or single L-restricted pnHSCs (**d**, n=17, 27-45 weeks post-primary transplantation) to the hematopoietic stem and progenitor cell hierarchy of primary recipients (abbreviations as defined in Supplementary information, Fig. S2). Grey denotes populations with no detectable donor contribution in any recipients, pink denotes contribution in some but not all recipients (frequency of reconstituted mice specified), and orange denotes contribution in all recipients.

Abbreviations: L, lymphoid (B and T cells); pnHSC, perinatal hematopoietic stem cell; PB, peripheral blood; SEM, standard error of the mean.
