# Supplementary material for: Platelet and myeloid lineage biases of transplanted single perinatal mouse hematopoietic stem cells
Source: Cell Res. 2023 Sep 6;33(11):883–6. doi: 10.1038/s41422-023-00866-4 (PMC10624660; doi:10.1038/s41422-023-00866-4)
Supplement: Supplementary file 7 — Supplementary information, Fig. S4 [file 41422_2023_866_MOESM7_ESM.pdf]

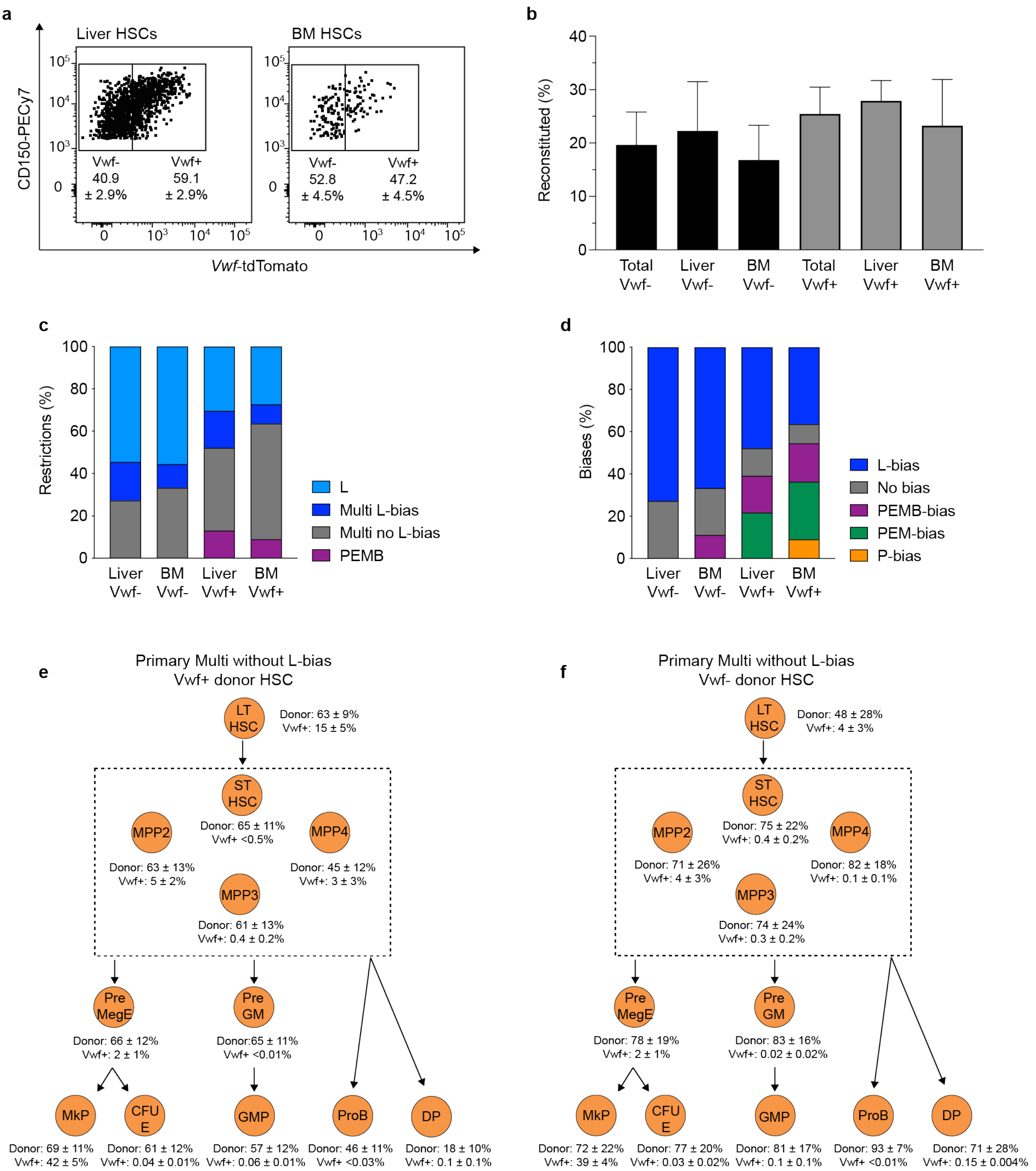

Supplementary information, Fig. S4: Comparison of lineage reconstitution patterns of *Vwf*-tdTomato^{-} and *Vwf*-tdTomato^{+} single pnHSCs from liver and BM.

a *Vwf*-tdTomato expression in liver and BM LSKCD150^{+}CD48^{-} pnHSCs. Representative flow cytometry profiles and mean ± SEM percentages of *Vwf*-tdTomato^{-} and *Vwf*-tdTomato^{+} pnHSCs are shown (n=7 for each tissue).

b Percentage (mean + SEM) of reconstituted mice (≥0.1% donor contribution to at least one PB lineage) at 25-26 weeks post-primary transplantation of single pnHSCs (liver *Vwf*-tdTomato^{-}, n=47; BM *Vwf*-tdTomato^{-}, n=55; liver *Vwf*-tdTomato^{+}, n=84; BM *Vwf*-tdTomato^{+}, n=65 transplanted recipients; 7 independent experiments). No statistically significant association between variables with Fisher's exact test (p=0.3433).

c-d Distribution of PB lineage restriction (**c**) and overall lineage bias (**d**) among reconstituted mice at 25-26 weeks post-primary transplantation (liver *Vwf*-tdTomato$^-$, n=11; BM *Vwf*-tdTomato$^-$, n=9; liver *Vwf*-tdTomato$^+$, n=23; BM *Vwf*-tdTomato$^+$, n=11). No statistically significant association between variables with Fisher's exact test (**c**, p=0.8164; **d**, p=0.2937).
e-f Mean (± SEM) donor contribution to the hematopoietic stem and progenitor cell hierarchy (abbreviations as defined in Supplementary information, Fig. S2) and *Vwf*-tdTomato expression in donor-derived compartments in the primary recipients represented in Supplementary information, Fig. S2c (27-49 weeks post-primary transplantation) when replenished by a single *Vwf*-tdTomato$^+$ (**e**, n=10) or *Vwf*-tdTomato$^-$ (**f**, n=3) multilineage pnHSC without L-bias. Index-sort information was not available for 1 of 14 pnHSCs in Supplementary information, Fig. S2c. Orange denotes contribution in all recipients
Abbreviations: pnHSC, perinatal hematopoietic stem cell; BM, bone marrow; LSK, Lineage$^-$Sca1$^+$Kit$^+$; SEM, standard error of the mean; PB, peripheral blood; L, lymphoid (B and T cells).
