# Supplementary material for: Platelet and myeloid lineage biases of transplanted single perinatal mouse hematopoietic stem cells
Source: Cell Res. 2023 Sep 6;33(11):883–6. doi: 10.1038/s41422-023-00866-4 (PMC10624660; doi:10.1038/s41422-023-00866-4)
Supplement: Supplementary file 8 — Supplementary information, Fig. S5 [file 41422_2023_866_MOESM8_ESM.pdf]

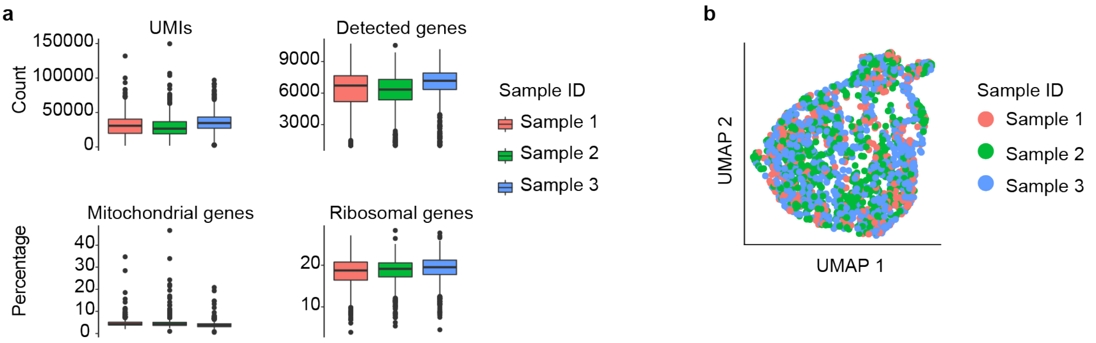

Supplementary information, Fig. S5: Quality control of single cell RNA sequencing data.

a Quality control metrics of sequenced LSKCD150^{+}CD48^{-} pnHSCs sorted from three female liver samples. Number of UMIs per cell in each sample, number of detected genes (non-zero expression in at least one sample) per cell in each sample, percentage of UMIs mapping to mitochondrial genes per cell, and percentage of UMIs mapping to ribosomal genes per cell are shown.

b UMAP of the sequenced LSKCD150^{+}CD48^{-} pnHSCs (n=1700 total single cells) visualized by sample ID.

Abbreviations: LSK, Lineage^{-}Sca1^{+}Kit^{+}; pnHSC, perinatal hematopoietic stem cell; UMI, unique molecular identifier; UMAP, uniform manifold approximation and projection.
