# Supplementary material for: Platelet and myeloid lineage biases of transplanted single perinatal mouse hematopoietic stem cells
Source: Cell Res. 2023 Sep 6;33(11):883–6. doi: 10.1038/s41422-023-00866-4 (PMC10624660; doi:10.1038/s41422-023-00866-4)
Supplement: Supplementary file 9 — Supplementary information, Fig. S6 [file 41422_2023_866_MOESM9_ESM.pdf]

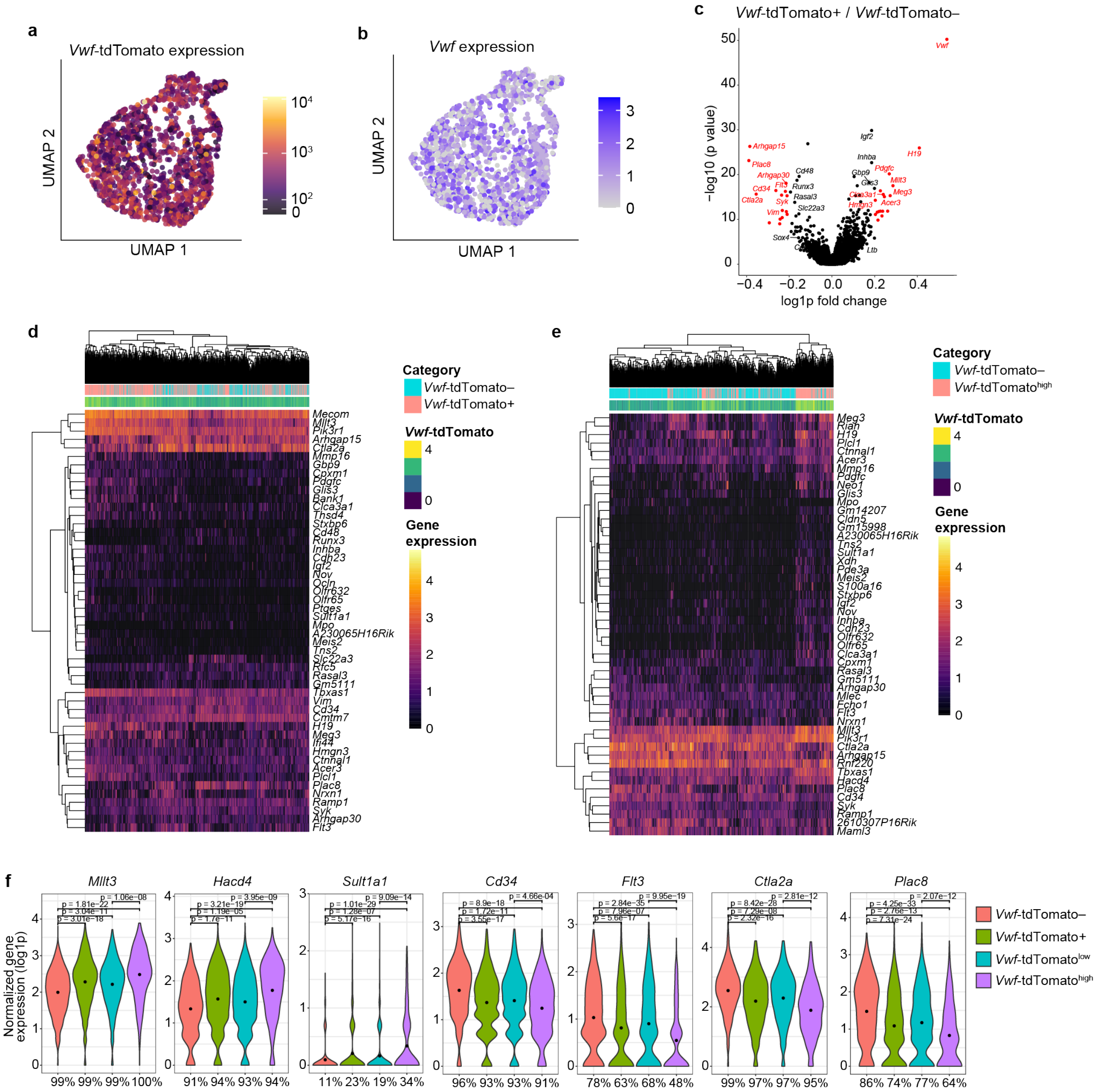


**Supplementary information, Fig. S6: Characterization of liver pnHSCs by single cell RNA sequencing.**

**a-b** UMAP of LSKCD150$^{+}$CD48$^{-}$ pnHSCs (n=1700 single cells from 3 liver samples) visualized by fluorescence level of *Vwf*-tdTomato (FACS index-sort) (**a**) and *Vwf* RNA expression (**b**).

**c** Volcano plot for DEGs (red; combined p-value <0.05) when comparing single *Vwf*-tdTomato$^{+}$ (n=572) with *Vwf*-tdTomato$^{-}$ (n=1128) pnHSCs. Downregulated genes on the left (log1p fold change <–0.2) and upregulated genes on the right (log1p fold change >0.2).

**d-e** Heatmap with hierarchical clustering based on the top 50 DEGs comparing *Vwf*-tdTomato$^{+}$ (n=572; **d**) or *Vwf*-tdTomato$^{high}$ (n=283; **e**) with *Vwf*-tdTomato$^{-}$ (n=1128) pnHSCs. First row shows binary classification into *Vwf*-tdTomato$^{-}$ (turquoise) and *Vwf*-tdTomato$^{+/high}$ (salmon) compartments and second row shows the relative fluorescence level of *Vwf*-tdTomato in each single cell as measured by FACS index-sort (purple=negative/low; yellow=high).

**f** Normalized expression (log1p) for selected DEGs (combined p-value <0.05) representing genes associated with adult lineage-restricted HSCs[1,2] in single *Vwf*-tdTomato$^{-}$ (n=1128), *Vwf*-

tdTomato$^+$ (n=572), *Vwf*-tdTomato$^{low}$ (n=289), and *Vwf*-tdTomato$^{high}$ (n=283) pnHSCs. Mean expression is represented by dots within violin plots and percentage of cells with detectable expression is indicated below. P-values represent combined Wilcoxon rank-sum test to test for difference in expression magnitude combined with Fisher's exact test to test for difference in expression frequency.
Abbreviations: pnHSC, perinatal hematopoietic stem cell; UMAP, uniform manifold approximation and projection; LSK, Lineage$^-$Sca1$^+$Kit$^+$; DEG, differentially expressed gene.

**REFERENCES**


1 Rodriguez-Fraticelli, A. E. *et al.* Single-cell lineage tracing unveils a role for TCF15 in haematopoiesis. *Nature* **583**, 585-589 (2020). https://doi.org:10.1038/s41586-020-2503-6

2 Pei, W. *et al.* Resolving Fates and Single-Cell Transcriptomes of Hematopoietic Stem Cell Clones by PolyloxExpress Barcoding. *Cell stem cell* **27**, 383-395 e388 (2020). https://doi.org:10.1016/j.stem.2020.07.018
